# Necroptosis and immune infiltration in hypertrophic cardiomyopathy: novel insights from bioinformatics analyses

**DOI:** 10.3389/fcvm.2024.1293786

**Published:** 2024-06-14

**Authors:** Can Hou, Sifan Fei, Fang Jia

**Affiliations:** Department of Cardiovascular Medicine, The First People’s Hospital of Changzhou, The Third Affiliated Hospital of Soochow University, Changzhou, Jiangsu Province, China

**Keywords:** hypertrophic cardiomyopathy, necroptosis, immune infiltration, bioinformatics analysis, cardiovascular diseases

## Abstract

**Background:**

Hypertrophic Cardiomyopathy (HCM), a widespread genetic heart disorder, is largely associated with sudden cardiac fatality. Necroptosis, an emerging type of programmed cell death, plays a fundamental role in several cardiovascular diseases.

**Aim:**

This research utilized bioinformatics analysis to investigate necroptosis's implication in HCM.

**Methods:**

The study retrieved RNA sequencing datasets GSE130036 and GSE141910 from the Gene Expression Omnibus (GEO) database. It detected necroptosis-linked differentially expressed genes (NRDEGs) by reviewing both the gene set for necroptosis and the differently expressed genes (DEGs). The enriched signaling pathway of HCM was assessed using GSEA, while common DEGs were studied through Gene Ontology (GO) and Kyoto Encyclopedia of Genes and Genomes (KEGG) pathways. Concurrently, the Protein-Protein Interaction network (PPI) proved useful for identifying central genes. CIBERSORT facilitated evaluating the correlation between distinct immune cell-type prevalence and NRDEGs by analyzing immune infiltration patterns. Lastly, GSE141910 dataset validated the expression ranks of NRDEGs and immune-cell penetration.

**Results:**

The investigation disclosed significant enrichment and activation of the necroptosis pathway in HCM specimens. Seventeen diverse genes, including CYBB, BCL2, and JAK2 among others, were identified in the process. PPI network scrutiny classified nine of these genes as central genes. Results from GO and KEGG enrichment analyses showed substantial connections of these genes to pathways pertaining to the HIF-1 signaling track, necroptosis, and NOD-like receptor signaling process. Moreover, an imbalance in M2 macrophage cells in HCM samples was observed. Finally, CYBB, BCL2, and JAK2 emerged as vital genes and were validated using the GSE141910 dataset.

**Conclusion:**

These results indicate necroptosis as a probable underlying factor in HCM, with immune cell infiltration playing a part. Additionally, CYBB, BCL2, JAK2 could act as potential biomarkers for recognizing HCM. This information forms crucial insights into the basic mechanisms of HCM and could enhance its diagnosis and management.

## 1 Introduction

As a multifaceted, autosomal dominant cardiovascular disease, Hypertrophic Cardiomyopathy (HCM) often correlates with genetic mutations within genes that encode cardiac sarcomere proteins (1). Left ventricular hypertrophy is a defining trait of HCM and it requires the elimination of other underlying cardiac, systemic or metabolic diseases as they could also lead to significant hypertrophy (2). The occurrence of HCM ranges from 0.02 to 0.23% in adults, but the annual incidence rate of sudden cardiac death (SCD) among adult patients with HCM is estimated to be around 1% (3, 4). Numerous studies have reported an increased mortality risk associated with HCM within the affected demographics (5–7). Therefore, investigating the pathogenesis of HCM is crucial as it can provide insights into clinical diagnosis and effective treatment modalities.

Necroptosis differentiates itself as a unique mode of programmed cell death that deviates from both apoptosis and necrosis, owing to its regulation by several cytokines and pattern recognition receptors (PRRs) (8). Cells undergoing necroptosis exhibit plasma membrane rupture and overall swelling, including mitochondrial swelling, a distinctive feature from apoptosis (9). Most reported necrotic pathways involve activating death receptors (DRs) by tumor necrosis factor α (TNF-α), which leads to the downstream recruitment of proteins like TNF-receptor-associated death domain (TRADD), receptor-interacting protein kinases 3 (RIPK3), and receptor-interacting protein kinases 1 (RIPK1) (10, 11). As a result of intra-molecular auto and trans-phosphorylation of RIP1/RIP3, mixed lineage kinase domain-like protein (MLKL) is recruited, leading to membrane pore formation, subsequent disruption of membrane integrity, and ultimately necrosis. Currently, there is growing interest in research to dissect various mechanisms governing necroptosis, with recent studies highlighting the role of necroptosis, particularly RIPK3 pathway-mediated necroptosis, as a promising future treatment strategy for HCM (12–15). Zou et al. have presented that a novel necroptotic loop compromised by CB2R serves as a critical driving force of diabetic heart dysfunction by recruiting the coregulator BACH2 (previously unrecognized as a cardiomyocyte transcription factor) to transcriptionally repress the expression of necroptosis (16). Studies indicate that angiotensin-(AngII)-induced cardiac hypertrophy in wild type mice correlates with an up-regulation of RIPK1 and RIPK3 (14). However, evidence to support these findings in human samples is currently lacking.

A recent study established links between an escalated myocardial infiltration of CD3 cells and unfavorable clinical outcomes in HCM patients (17). Moreover, poor prognosis of HCM has also been linked to docosahexaenoic acid (DHA) intake, which is known to exacerbate heart failure (18). Early inflammation in HCM could potentially stem from cardiomyocyte disarray, sarcomere damage, mitochondrial oxidative stress, and microvascular dysfunction (19). However, understanding of how immune cells infiltrate HCM remains unclear.

Leveraging bioinformatics tools and the Gene Expression Database (GEO), this research aims to investigate the correlation between immune infiltration, necroptosis, and HCM progression. It also seeks to identify key genes associated with necroptosis in HCM and decode their functional mechanisms. Besides, it will explore the linkage between necroptosis and immune cell infiltration aiming to reveal potential correlations between these processes and the development of HCM.

## 2 Materials and methods

### 2.1 Acquiring and processing RNA sequencing dataset

**Figure 1:**
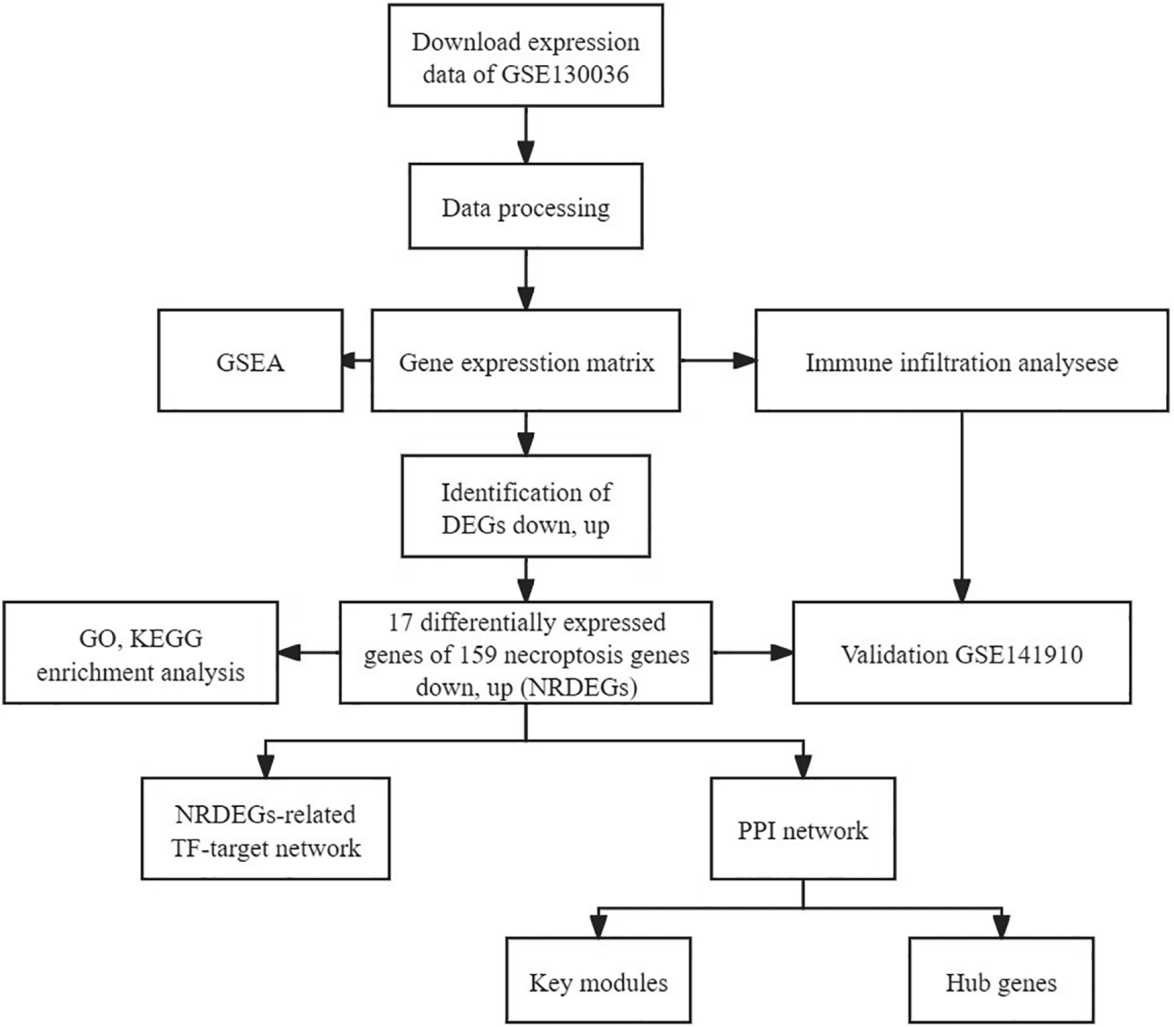
Flowchart depicting the multistep screening strategy used to analyze the bioinformatics data.

The publicly accessible Gene Expression Omnibus (GEO) database (https://www.ncbi.nlm.nih.gov/geo/) provides microarray data for various disease investigations. RNA-seq transcriptome data alongside clinical details of HCM patients were sourced from GEO, utilizing the “GEO query” R package. The GSE130036 dataset, annotated on the GPL20795 platform, encompassed tissue samples from 28 HCM patients (9 females and 19 males; average age: 33.4 ± 8.2 years) and 9 healthy individuals (one female and eight males; average age: 37.7 ± 6.2 years). The “Deseq2” R package facilitated the detection of differentially expressed genes between HCM and healthy myocardium tissues (20, 21). Furthermore, to validate these findings externally, we utilized the GSE141910 RNA sequencing dataset (comprising 28 HCM left ventricle tissues from 11 females and 17 males with an average age of 48.7 ± 12.6 years and 166 normal left ventricle tissues from 89 females and 77 males with an average age of 55.9 ± 14.0 years). Heatmaps were generated using R software. All study data was obtained from GEO and hence did not necessitate obtaining ethical approval or informed consent. Figure 1 shows the workflow chart adopted in this study.

### 2.2 Selection of genes associated with necroptosis

“Deseq2” R package (version 1.38.3) was employed to ascertain differentially expressed genes (DEGs), with a cut-off criteria set at |Log2-fold change| > 0.5 and *P*-value < 0.05 (22). DEGs were visually represented using volcano plots and heatmaps constructed via the ggplot2 and heatmap R packages, respectively. Necroptosis-related genes were identified from the M24779.gmt gene set sourced from the Gene Set Enrichment Analysis (GSEA) tool (http://www.gsea-msigdb.org/gsea/index.jsp). This gene set comprised 159 necroptosis-associated genes as annotated by the Kyoto Encyclopedia of Genes and Genomes (KEGG) Pathways databases. By comparing DEGs with the necroptosis gene set, we uncovered differentially expressed necroptosis genes (NRDEGs). The Venn diagram package was used to execute this analysis.

### 2.3 Protein-protein interaction (PPI) network analysis and hub gene identification

We utilized the Search Tool for the Retrieval of Interacting Genes/Proteins (STRING; https://string-db.org/) to develop the Protein-Protein Interaction (PPI) network (22). The resulting network data was visualized on Cytoscape (23). To identify hub genes and significant modules within the PPI network, we employed CytoHubba plugin in Cytoscape and used its MCC algorithm (24). We identified significant modules within the network using the MCODE plugin in Cytoscape, with the following parameters: degree cut-off = 2, node score cut-off = 0.2, k-core = 2, and max depth = 100 (25).

### 2.4 The gene set enrichment analysis

In this study, gene expression enrichment analysis was conducted using three approaches: Gene Ontology (GO) enrichment analysis, KEGG pathway analysis, and Gene Set Enrichment Analysis (GSEA). These analyses identified functional annotations, significant biological pathways, and enriched gene sets associated with differentially expressed genes (26–28). This was conducted with the aid of the R package ClusterProfiler, which facilitated the recognition of potential biological functions and pathways associated with HCM and normal controls (29). Enriched pathway results were considered statistically significant if the false discovery rate (FDR) was below 0.05. GSEA analysis was conducted using the “clusterProfiler” package in the molecular signature database (MSigDB). A specified number of sample permutations (1,000) and a significance level of *P* < 0.05 were utilized to investigate the potential mechanisms associated with the c2 (c2.cp.kegg.v7.5.1.entrez.gmt) and c5 (c5.bp.v7.5.1.entrez.gmt) gene sets (30). Lastly, Gene set variation analysis (GSVA) was conducted to explore potential associations between biological pathways and gene signatures, estimating pathway activity scores based on gene expression data (31).

### 2.5 Validation of diagnostic values of hub genes

We utilized mRNA expression data from GSE130036 to construct receiver operating characteristic (ROC) curves using the “pROC” package. The area under the curve (AUC) was calculated for the hub gene, indicating its diagnostic efficiency. A higher AUC value indicates a higher efficiency in diagnosing the gene. Additionally, this result was further validated using dataset GSE141910.

### 2.6 Assessing immune cell infiltration

The CIBERSORT algorithm, which can be found at http://cibersort.stanford.edu, is a highly precise calculation method that utilizes deconvolution to accurately determine the compositions of various immune cell subtypes. By utilizing CIBERSORT, the unique characteristics of each immune cell subtype can be effectively displayed, providing valuable insights into the immune system (32). A computational method was employed in this study to estimate the abundance of 22 distinct immune cell types through 1,000 permutations by the validated leukocyte gene signature matrix (LM22) (32). The complete list of genes (LM22) utilized to identify immune cells is available in the Supplementary Presentation 1. To examine the differences between various immune cell types, we employed the Wilcoxon rank-sum test. Additionally, to investigate the relationship between NRDEGs and different immune cell types, we conducted Spearman correlation analysis.

### 2.7 Confirmation of NRDEGs and immune infiltration in GSE141910

Statistical significance was set at a threshold of *P* < 0.05 when comparing the expression of NRDEGs between HCM and normal samples using the GSE141910 dataset. Results were visualized using the ggpurb and ggplot2 packages in R. We also estimated immune cell abundance in the GSE141910 dataset using CIBERSORT. Moreover, identified hub genes were subjected to ROC analysis to verify their accuracy.

## 3 Results

### 3.1 DEGs and NRDEGs identification and analysis

**Figure 2:**
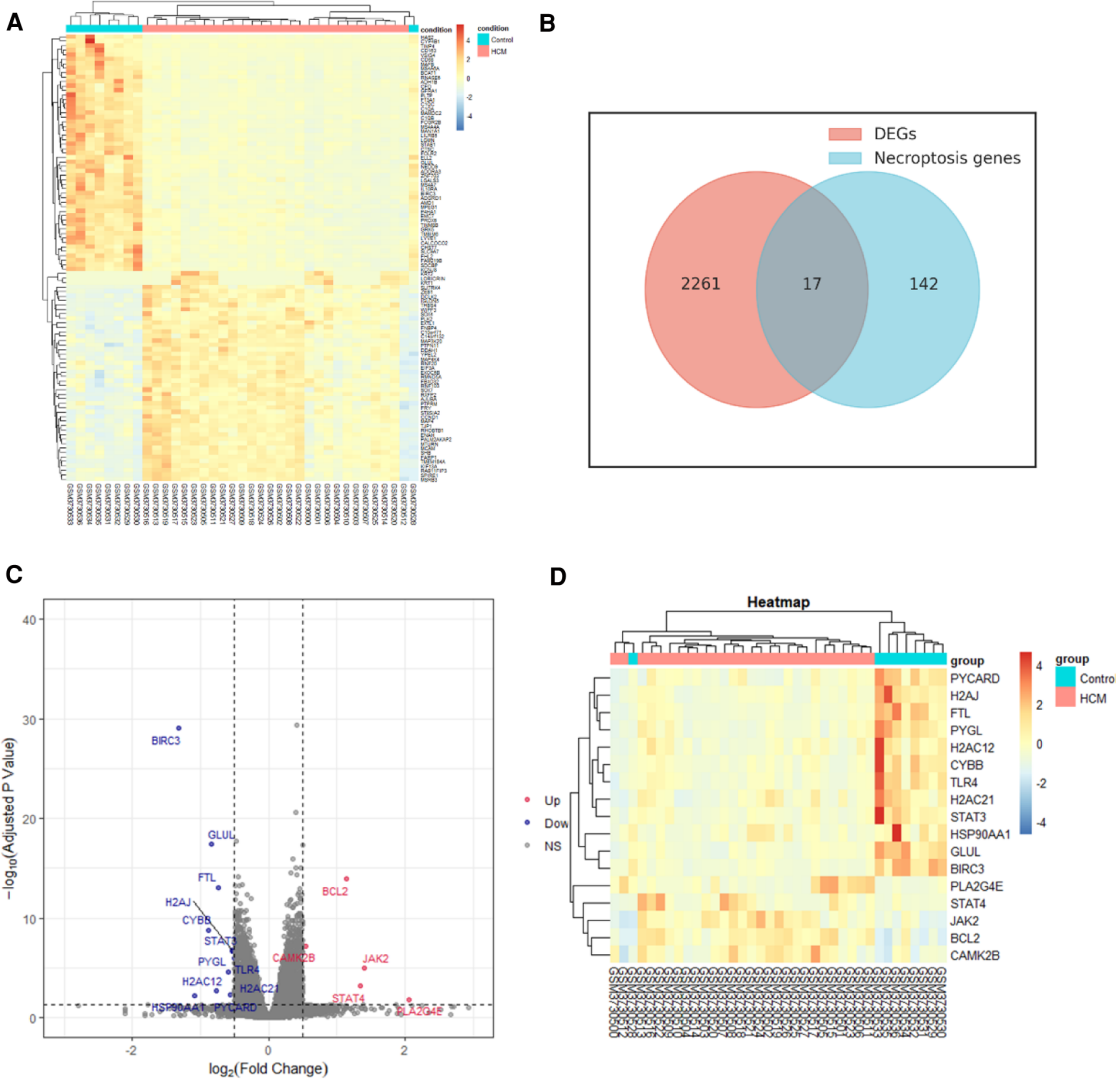
Identification of differentially expressed necroptosis-related genes (NRDEGs) in HCM. (**A**) Heatmap depicting the clustered analysis of DEGs in GSE130036 dataset. (**B**) Venn diagram illustrating the overlap between DEGs in GSE130036 and necroptosis-related genes in KEGG pathway databases. (**C**) Volcano plot displaying NRDEGs. (**D**) Heatmap showing the clustered analysis of NRDEGs.

We identified 2,278 DEGs between HCM and normal tissues through the analysis of GSE130036 dataset. Based on the expression levels of each gene, a heatmap was generated (Figure 2A). Out of the 2,278 DEGs, 1,078 were upregulated while 1,200 were downregulated. Among the 17 overlapping genes identified between the DEGs and NRDEGs, a total of 5 genes were upregulated, while the remaining 12 genes were downregulated in HCM samples compared to normal samples. The Venn diagram analysis highlighted 17 overlapped genes as NRDEGs, including PYGL and BCL2 (Figure 2B). Volcano plots and heatmaps (Figures 2C,D) were generated to visualize NRDEGs. The complete list of genes overlapping among the DEGs and NRDEGs is provided in the Supplementary Presentation 1.

### 3.2 Gene set enrichment analysis (GSEA) profile

**Figure 3:**
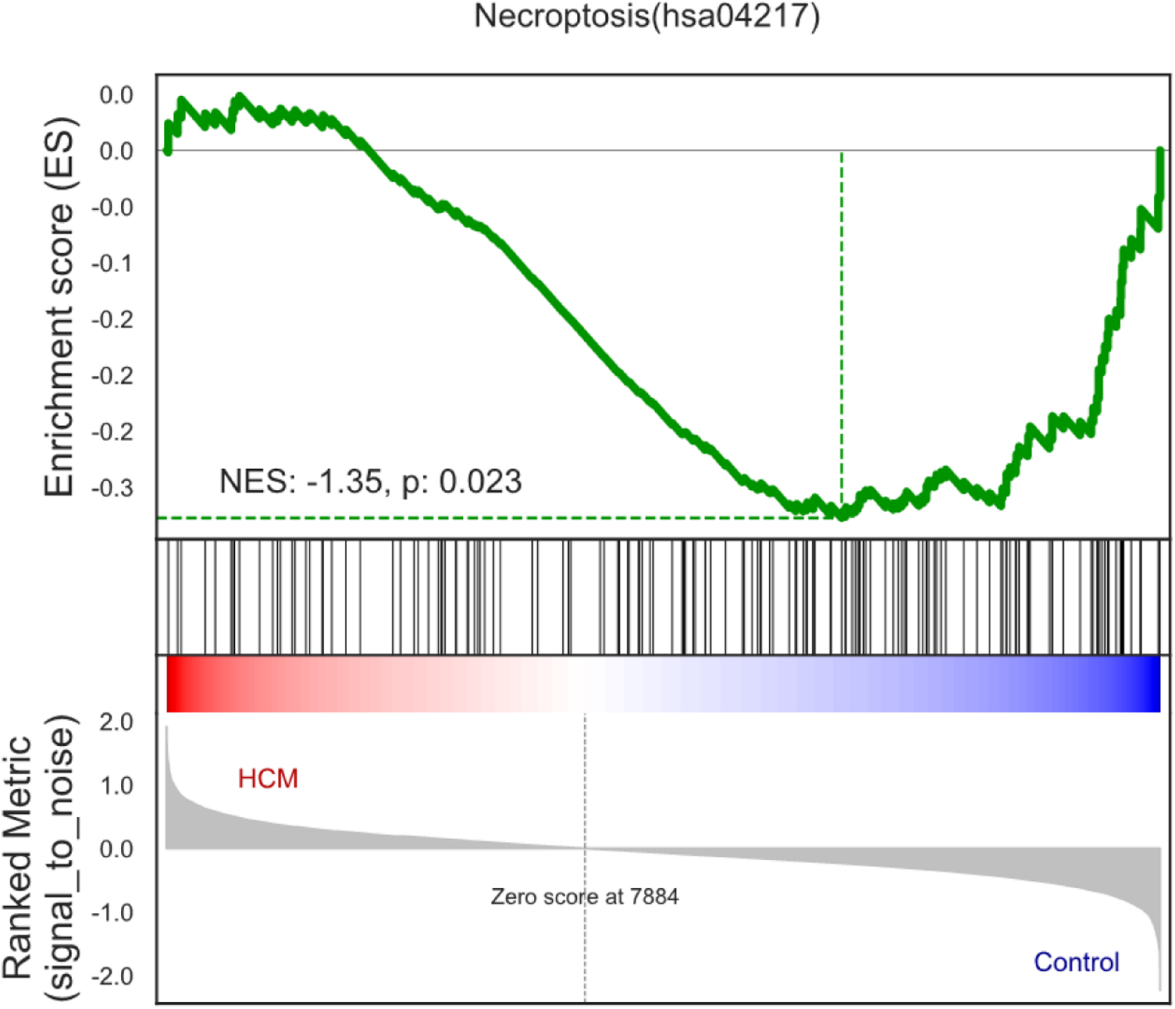
Enrichment plots of gene set enrichment analysis in HCM.

For the purpose of identifying the biological pathways associated with DEGs in HCM, we performed GO and KEGG database searches. According to Figure 3, necroptosis was significantly upregulated in HCM samples (normalized enrichment score = −1.35, *P*-value = 0.023), suggesting a close association between HCM and necroptosis.

### 3.3 Exploring the underlying functional and mechanism of NRDEGs

**Figure 4:**
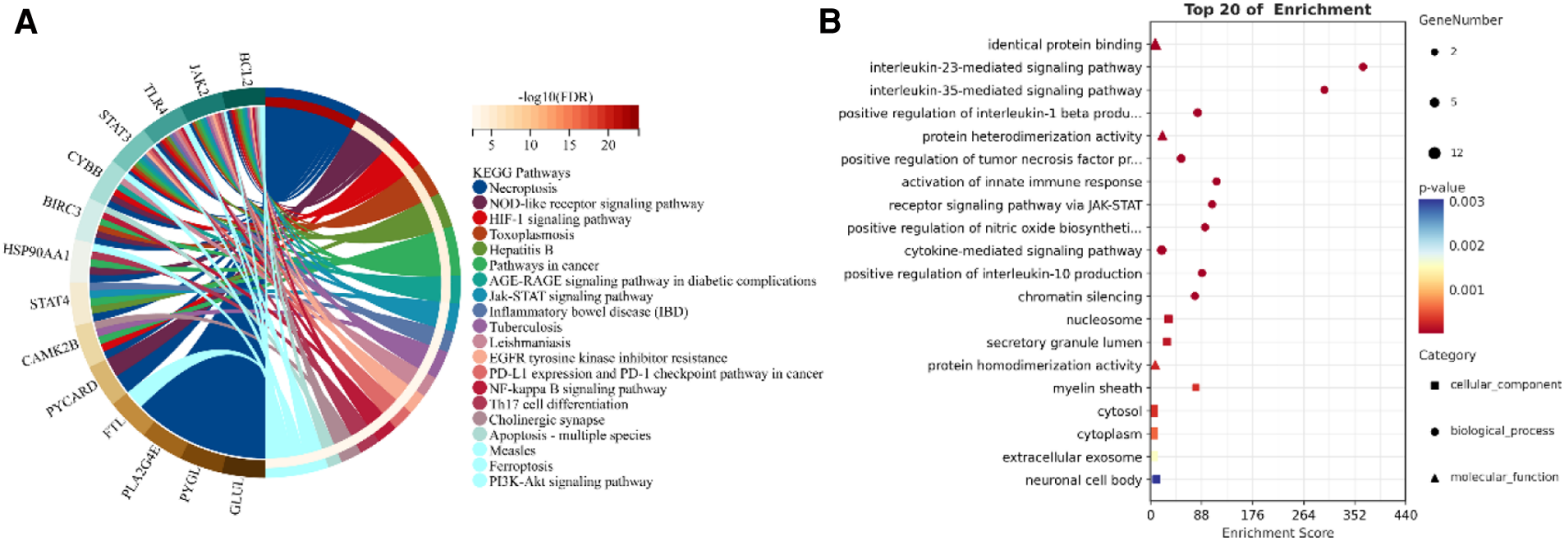
Enrichment analysis of NRDEGs. (**A**) GO enrichment terms obtained from the analysis. (**B**) Top 20 pathways identified in KEGG pathways.

Through a KEGG and GO enrichment analysis of NRDEGs, we identified several biological functions and signaling pathways closely linked to HCM (Figures 4A,B). Our KEGG pathway enrichment analysis revealed the top 20 pathways with significant enrichment of differential genes, which include necroptosis, NOD-like receptor signaling pathway, hypoxia-inducible factor- (HIF-)1 signaling pathway, toxoplasmosis, AGE-RAGE signaling pathway in diabetic complications, Jak-STAT signaling pathway, and tuberculosis. Our BP analysis showed significant enrichment in the interleukin- (IL-) 23-mediated signaling pathway, IL-35-mediated signaling pathway, activation of innate immune response, and receptor signaling pathway via JAK-STAT. In the CC analysis, the NRDEGs were highly enriched in the myelin sheath, nucleosome, and secretory granule lumen. Moreover, the MF analysis indicated significant enrichment in protein heterodimerization activity, protein homodimerization activity and identical protein binding. The comprehensive table displaying the KEGG and GO pathways resulting from our analyses is provided in the Supplementary Presentation 1.

### 3.4 PPI network analysis and hub gene identification

**Figure 5:**
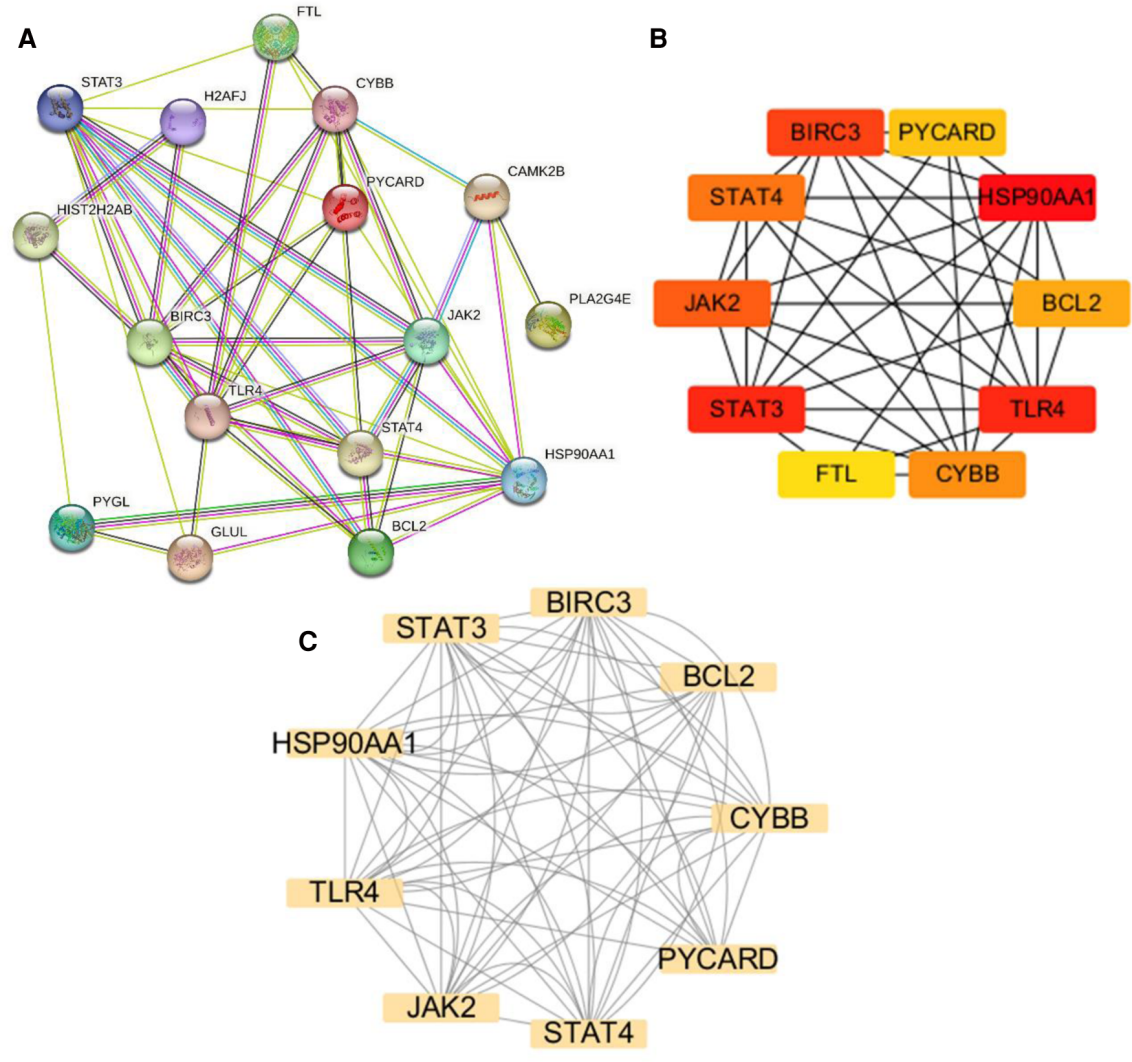
Protein-protein interaction (PPI) networks and key modules in HCM. (**A**) PPI network constructed using STRING and visualized in Cytoscape. (**B**) Identification of the top 10 hub genes using CytoHubba. (**C**) Network clustering analysis performed using Molecular Complex Detection.

The NRDEGs PPI network was constructed with the STRING database (Figure 5A) and analyzed using Cytoscape software. The top 10 hub genes were identified using the CytoHubba MCC algorithm, which included the baculoviral IAP repeat-containing 3 (BIRC3), signal transducer and activator of transcription 3 (STAT3), STAT4, heat shock protein 90 alpha family class A member 1 (HSP90AA1), toll-like receptor 4 (TLR4), janus kinase-2 (JAK2), PYD and CARD domain containing (PYCARD), CYBB, B-cell lymphoma 2 (BCL2) and Ferritin Light Chain (FTL) (Figure 5B). The MCODE plugin in Cytoscape was utilized to identify significant gene clusters, and the cluster scores were calculated. The results, including the identified clusters, are presented in Figure 5C. This module was composed of nine potential hub genes, namely HSP90AA1, CYBB, STAT3, BIRC3, BCL2, PYCARD, STAT4, JAK2, and TLR4 (Figure 5C). By intersecting the hub genes identified by both CytoHubba MCC and MCODE, we obtained a list of nine hub genes, including HSP90AA1, CYBB, STAT3, BIRC3, BCL2, PYCARD, STAT4, JAK2, and TLR4.

### 3.5 ROC curve analysis

**Figure 6:**
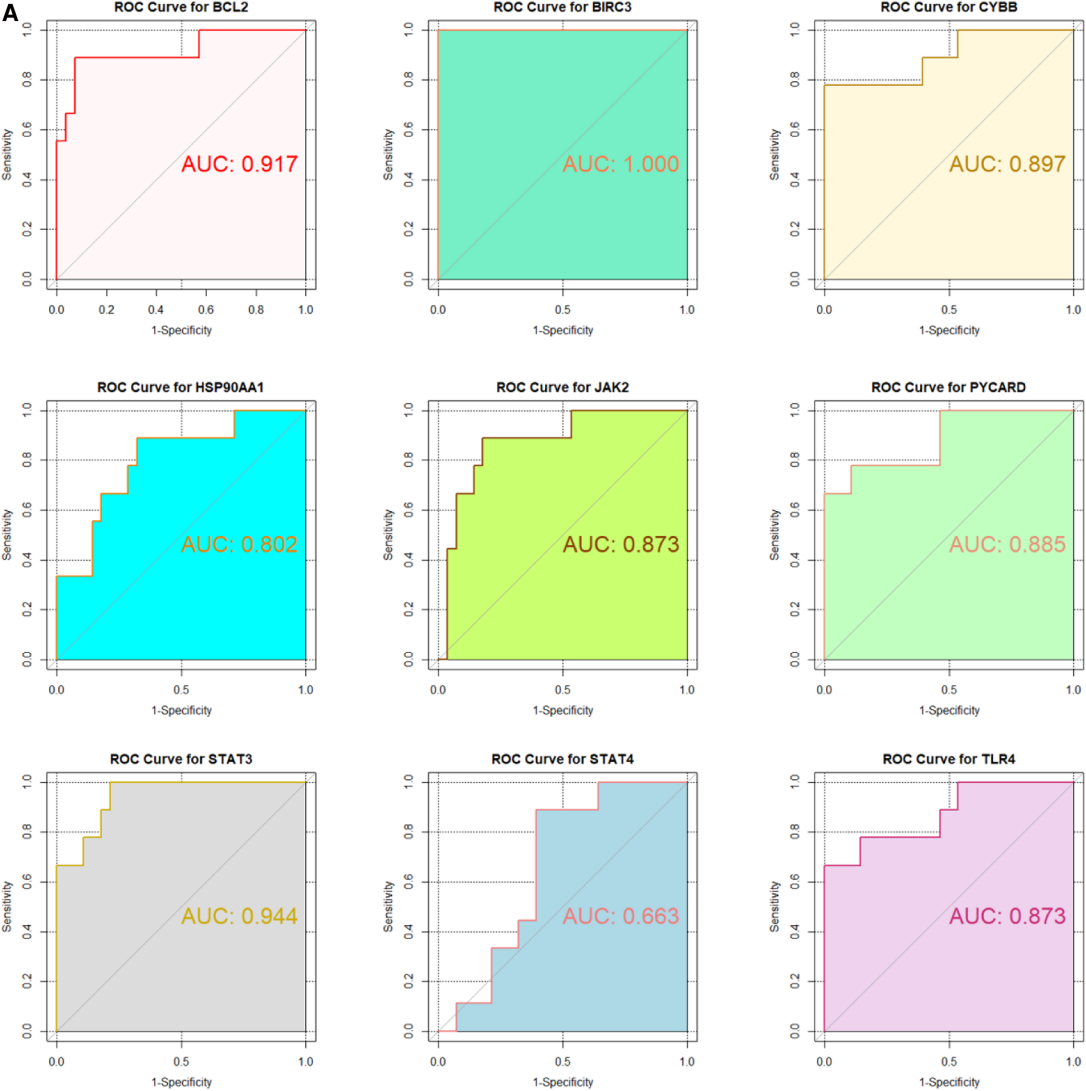

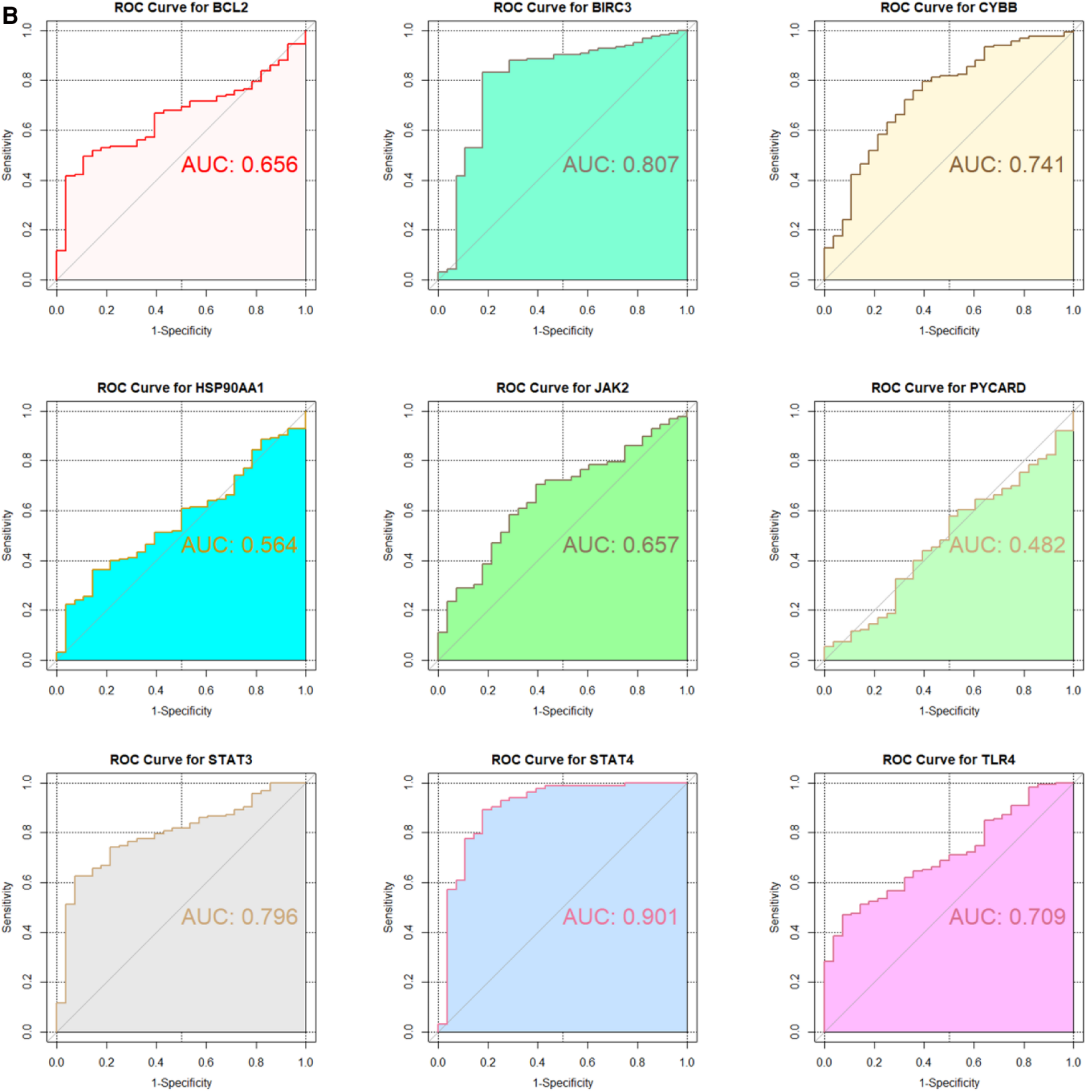
Validation of candidate Hub genes by ROC curve analysis. (**A**) ROC curve analysis of hub genes in the GSE130036 dataset. (**B**) ROC curve analysis of hub genes in the GSE141910 dataset, serving as the validation set.

In the GSE130036 and GSE141910 datasets, to explore the accuracy of the 9 hub genes as the diagnostic biomarkers for HCM, the ROC curves were plotted (Figures 6A,B). Among these 9 hub genes, the AUC values for 7 genes, namely BIRC3, BCL2, CYBB, STAT4, JAK2, TLR4, and STAT3, exceeded 0.6 in both datasets. This indicates that these hub genes exhibit substantial diagnostic potential in HCM (Figures 6A,B).

### 3.6 Different immune cell types analysis between HCM and control group

**Figure 7:**
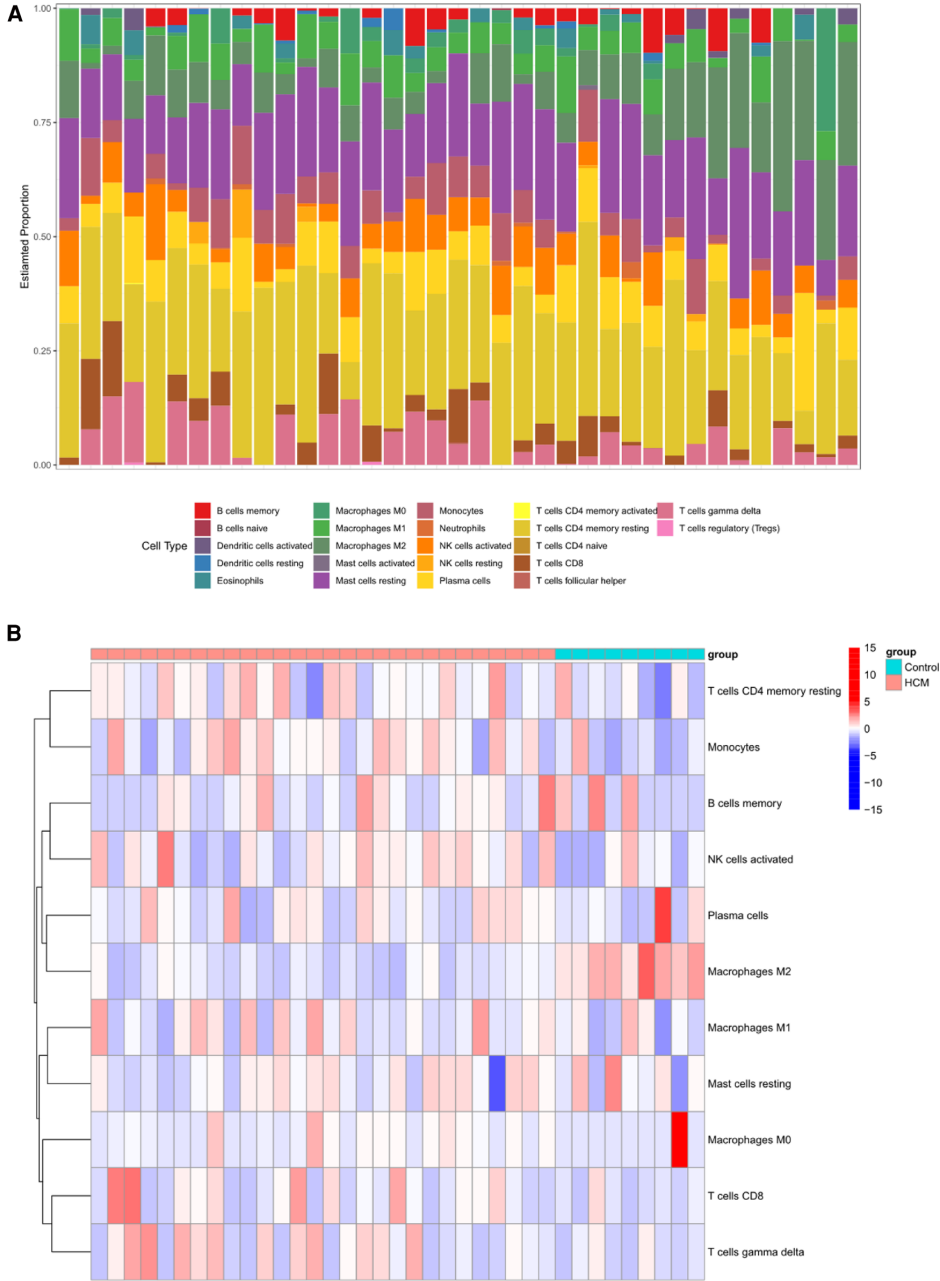

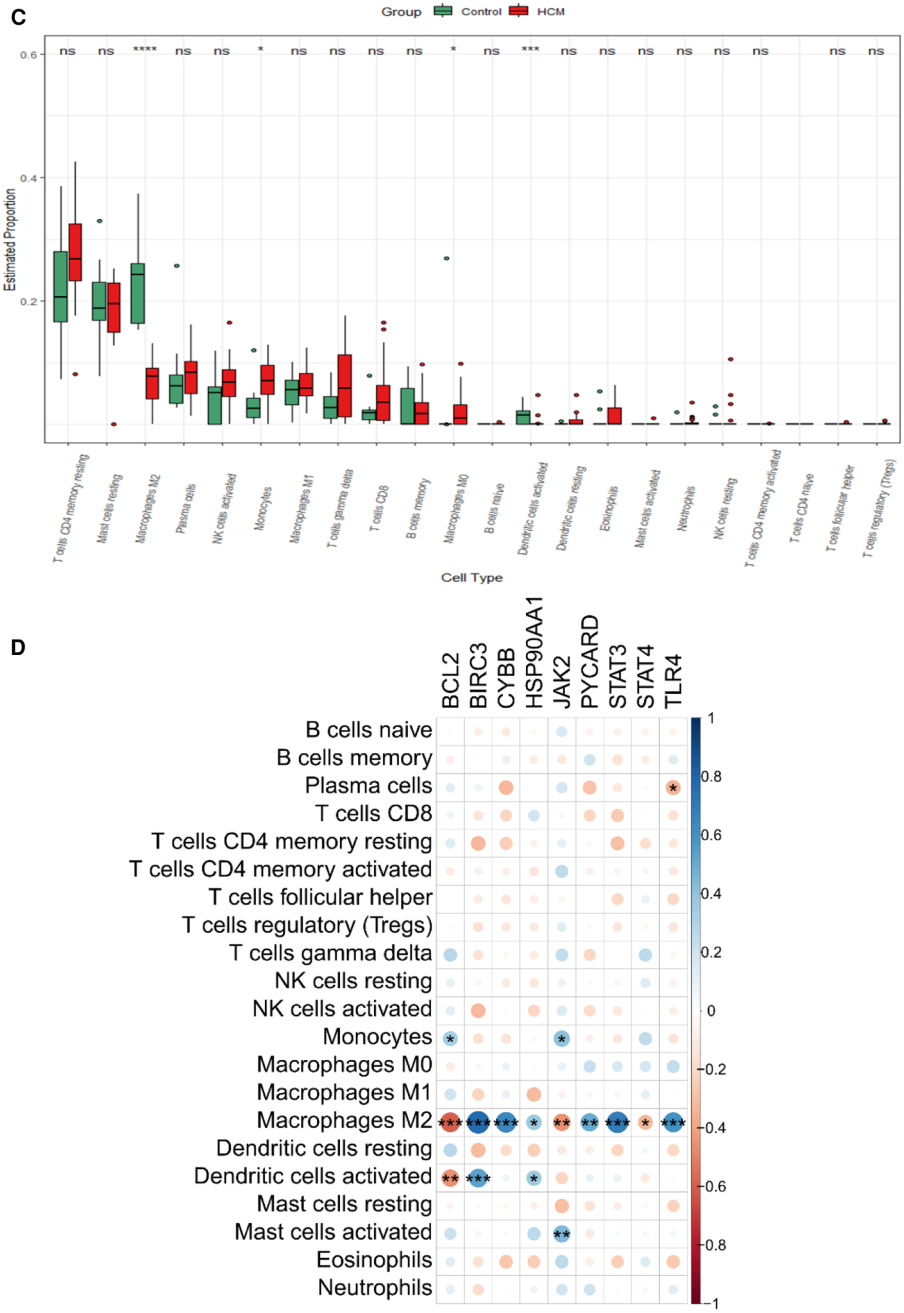
Immune infiltration landscape in HCM. (**A**) Stacked bar chart illustrating the proportions of different immune cell types. (**B**) Heatmap displaying the proportions of immune cell types. (**C**) Violin plot showing the distribution of immune cell proportions. (**D**) Correlation analysis between differentially expressed necroptosis-related hub genes and immune cells. *P < 0.05, **P < 0.01, ***P < 0.001, ****P < 0.0001.

Mounting evidence suggests a close association between HCM and the immune microenvironment. In this study, we investigated different immune cell types in GSE130036. CIBERSORT analysis was conducted on all the samples of GSE130036, allowing us to determine the proportions of different immune cells. The histogram represents these proportions using different colors (Figure 7A). The results indicate that monocytes, resting mast cells, regulatory T cells (Tregs), activated natural killer (NK) cells, resting NK cells, M2 macrophages, resting memory CD4T cells, naive B cells, and eosinophils may represent the predominant immune cells in the myocardial tissue of HCM patients (Figure 7B). Compared with controls, the abundance of M2 macrophages and Dendritic cells resting markedly decreased in HCM patients, while the abundance of monocytes and M0 macrophages were up-regulated (Figure 7C). The majority of NRDEGs showed correlations with M2 macrophages (Figure 7D). Specifically, BCL2, JAK2, and STAT4 were positively correlated with M2 macrophages, while BIRC3, CYBB, HSP90AA1, PYCARD, STAT3, and TLR4 were negatively correlated with M2 macrophages.

### 3.7 Validation of hub gene expression and analysis of immune cell types in HCM

**Figure 8:**
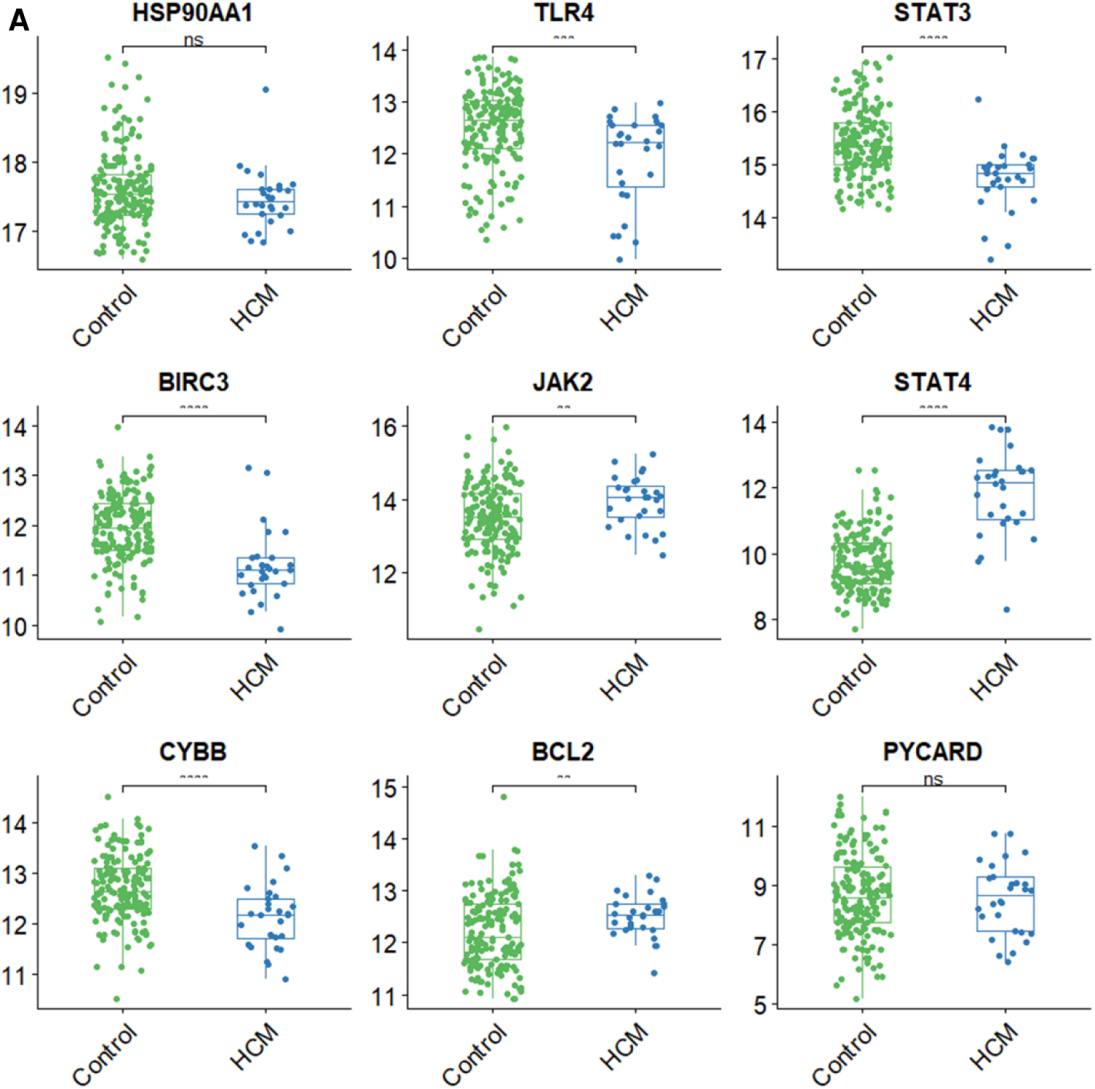

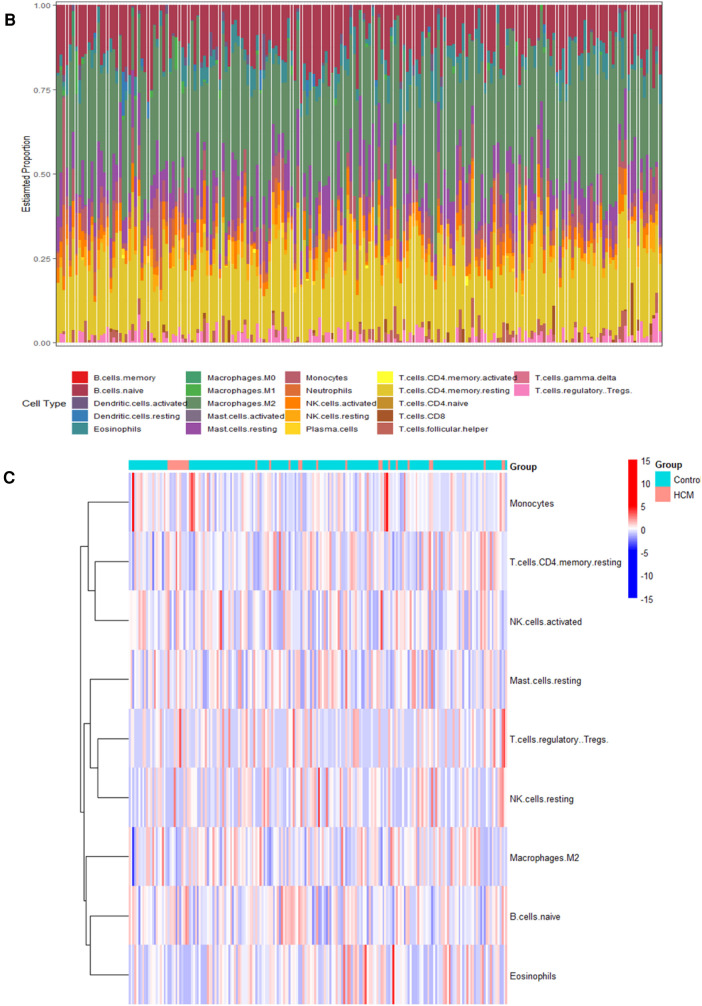

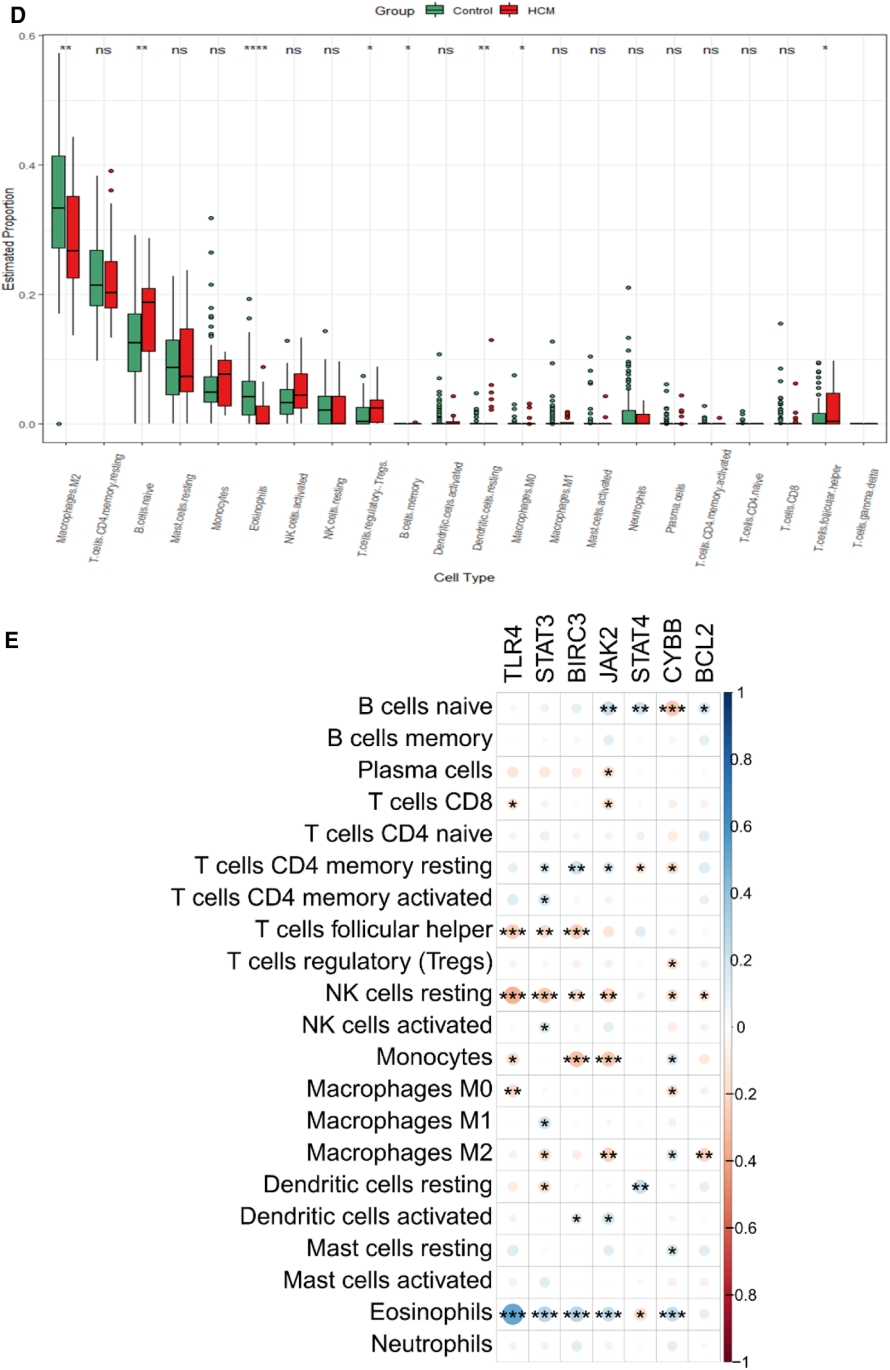
Validation of NRDEGs and immune infiltration in HCM. (**A**) Expression levels of the 9 hub NRDEGs in the GSE141910 dataset. (**B**) Stacked bar chart illustrating the proportions of different immune cell types. (**C**) Heatmap displaying the proportions of immune cell types. (**D**) Violin plot depicting the distribution of immune cell proportions. (**E**) Correlation analysis between NRDEGs and immune. *P < 0.05, **P < 0.01, ***P < 0.001, ****P < 0.0001.

To validate the findings, a dataset independent of and GSE141910 (166 normal myocardial tissues and 28 HCM tissues) was used. Among the nine hub genes, including BIRC3, BCL2, CYBB, PYCARD, STAT4, JAK2, TLR4, HSP90AA1, and STAT3, extracted expression data revealed that seven of them (BIRC3, BCL2, CYBB, STAT4, JAK2, TLR4 and STAT3) showed consistent trends with the findings from GSE130036 dataset (Figure 8A). Our analysis revealed that the HCM group showed immune cell infiltration primarily composed of T cells CD4 memory resting, M2 macrophages, CD8 naive cells and B cells memory (Figures 8B,C). Significant differences were observed in the abundance of 8 immune cell types between HCM and control samples (Figure 8D). The validated hub genes JAK2, CYBB, and BCL2 exhibited a significant correlation with the infiltration of M2 macrophages by Spearman analysis of the relationship between validated hub genes and immune cell types (Figure8E). Notably, these results were consistently observed in both the training and validation sets.

## 4 Discussion

Necroptosis has been verified to participate in various cardiovascular diseases, such as atherosclerosis, myocardial infarction, myocarditis, cardiac ischemia/reperfusion (I/R) injury, and others (33). However, limited research exists on gene biomarkers associated with immune infiltration and necroptosis in hypertrophic cardiomyopathy (HCM) compared to normal tissues. In our study, we identified 17 NRDEGs. Through analysis of the GSE1360036 dataset's PPI network, we pinpointed nine hub genes: HSP90AA1, CYBB, STAT3, BIRC3, BCL2, PYCARD, STAT4, JAK2, and TLR4. GSEA analysis exposed several significant biological pathways potentially contributing to HCM development. Our scrutiny of two GEO datasets unveiled CYBB, BCL2, and JAK2's possible diagnostic value in HCM. Interestingly, pronounced differences exist in immune cell composition between HCM and normal samples. Specifically, CYBB showed a positive correlation with M2 macrophages, while BCL2 and JAK2 exhibited negative correlations. Investigating the mechanisms underlying the involvement of key immune cells and pathways in cardiomyopathy pathogenesis can provide valuable insights into the role of the immune system in regulating cardiac function. Additionally, it may help identify potential targets for cardiovascular immunotherapy.

The inflammatory response is crucial for promoting adaptive remodeling after cardiac injury. However, inflammation can impair adaptive responses and worsen cardiac injury (34). Cardiac macrophages divide into two subpopulations: pro-inflammatory M1 and reparative M2 macrophages. Each plays distinctive roles in cardiac inflammation. M1 macrophages partake in the acute inflammatory response, secreting cytokines like TNF-α, IL-1β, and IL-6. Conversely, M2 macrophages possess anti-inflammatory properties, producing cytokines such as VEGF and TGF-β. These cytokines contribute to cardiac fibroblast (CF) activation, angiogenesis, and wound healing (35). *In vivo* studies have shown that administration of IL-10 can enhance the expression of M2 marker genes, activate cardiac fibroblasts (CFs), and ultimately lead to improved structural and functional cardiac remodeling. Additionally, M2 macrophages inhibit maladaptive ventricular remodeling and promote cardiomyocyte proliferation and angiogenesis after heart injury in newborn mice (36). Cardiac M2 macrophages are not only critical for maintaining heart homeostasis but also play a key role in heart repair and regeneration following injury (37). Given the significant presence of macrophages in cardiac tissue and their essential functions, it is important to further investigate their phenotypes, functions, and dynamics under both normal and disease conditions. Such research will advance our understanding of macrophage biology in the heart and its implications for cardiovascular health and disease.

JAK2, part of the Janus kinase (JAK) family, emerged as a key gene in our study, and its high expression in HCM implies its significance in disease pathology. Previous research has shown that JAK2 kinase activation, along with the activation of BAX, caspase-1, and caspase-2 activity, is associated with apoptosis induced by angiotensin II in cultured adult cardiomyocytes (38). Numerous studies have indicated that inhibiting JAK2 can effectively inhibit apoptosis and its associated signaling pathways, providing potential therapeutic implications for cardiac pathologies (38). Additionally, JAK2 inhibition has been shown to protect against ischemic and reperfusion injury in rat hearts and prevent the opening of mitochondrial transition pores induced by leptin. The JAK2-V617F somatic mutation, associated with excessive JAK2 activation in myeloproliferative diseases, has also been reported in a suspected case of HCM, highlighting the importance of investigating the role of JAK2 in HCM (39, 40). The Jak2 gene has previously been identified as a hallmark gene in HCM, as reported in the literature (41). Studies have demonstrated upregulation of JAK2 transcriptional regulation independent of the JAK2-V617F mutation in confirmed cases of HCM. This upregulation is associated with increased expression of JAK2 and its canonical target, STAT3, in the left ventricle and cardiomyocyte nuclei, implicating dysregulation of the JAK2-STAT3 pathway in HCM pathogenesis (42). The upregulation of JAK2 in HCM may be explained by increased bioactivity of IL-6 and oxidative stress frequently observed in this disease. IL-6 and oxidant stress could contribute to dysregulation of JAK2 expression, but additional studies are needed to validate and confirm this hypothesis (43).

BCL2 is a regulatory protein from the BCL family involved in apoptosis control. It can play dual roles as proapoptotic or antiapoptotic. It can act as either a proapoptotic or antiapoptotic protein, exerting opposing effects on the induction or inhibition of apoptosis, respectively (44). Its primary function is to maintain cell survival and prevent excessive cell death. During necroptosis, receptor-interacting protein kinase 1 (RIPK1) and RIPK3 form a complex called the necrosome, which triggers necroptotic signaling. In this process, BCL2 can have a dual role. On one hand, increased levels of BCL2 can inhibit necrosome formation and suppress necroptosis by sequestering RIPK1 and RIPK3. This anti-necroptotic function of BCL2 is often referred to as “necroptosis suppression” (45). On the other hand, under specific circumstances, BCL2 can also promote necroptosis. When caspase-8, a key mediator of apoptosis, is inhibited or not fully activated, it fails to cleave and inactivate RIPK1. Uninhibited RIPK1 then recruits RIPK3 to form the necrosome, leading to necroptotic cell death (46). BCL2 can facilitate this process by indirectly promoting RIPK1/RIPK3-mediated necroptosis through its anti-apoptotic activity (47). ErbB2 over-expression also leads to up-regulation of the pro-survival bcl-2 family of proteins in the heart, with an anti-apoptotic shift in the balance of pro-survival bcl-xL and apoptotic bcl-xS proteins (48). Therefore, BCL2 acts as a critical regulator in the decision-making process between apoptosis and necroptosis. Depending on the context and cellular conditions, BCL2 can either inhibit apoptosis or modulate the switch towards necroptosis. The interplay between BCL2 and the signaling pathways governing apoptosis and necroptosis is still an active area of research, and the precise mechanisms underlying this relationship are not fully understood. Previous studies have highlighted the significant contribution of BCL2 in the context of cardiac hypertrophy (49, 50). Inhibitors of BCL2, such as LY294002 (which inhibits the PI3K/AKT signaling pathway) and Venetoclax (a selective BCL2 inhibitor), have been shown to possess protective effects against cardiac hypertrophy by inhibiting BCL2 (51). The study highlights the role of BCL2 as a key downstream effector in the PI3K-Akt signaling pathway, which is crucial for maintaining cardiomyocyte survival and promoting cardiac hypertrophy (51). This study suggests the potential involvement of BCL2 in HCM development. However, additional comprehensive research is needed to fully understand the underlying mechanisms by which BCL2 contributes to HCM. Further investigations will help elucidate the role of BCL2 in HCM pathology and may provide insights into potential therapeutic strategies for managing this condition.

The CYBB gene, alternatively known as NOX2, encodes a variant of Cytochrome b serving as the terminal element of the respiratory chain. It facilitates the transfer of single electrons from cytoplasmic nicotinamide adenine dinucleotide phosphate (NADPH) across the plasma membrane to external molecular oxygen. CYBB is expressed in various cell types, including eosinophils, neutrophils, B lymphocytes, and other cell lineages. Its main function is to generate reactive oxygen species (ROS) in response to cytokines and stimuli such as IFN-*γ*, lipopolysaccharide (LPS), and TNF-α (52). Indeed, CYBB plays a critical role as the central component of the microbicidal oxidase system in phagocytes. It is responsible for generating ROS involved in microbe killing. Importantly, CYBB is not limited to hematopoietic cell lineages such as neutrophils and eosinophils but is also expressed in non-hematopoietic cell lineages, including fibroblasts, endothelial cells, and cardiomyocytes. This highlights the broad involvement of CYBB-mediated ROS production in various cellular processes beyond immune responses (53). CYBB upregulation in hypertrophy has been proposed as a potential diagnostic biomarker for HCM and its involvement in maladaptive processes (54, 55). CYBB activation in dietary obesity and metabolic disorders contributes to cardiac oxidative stress, abnormal redox signaling, and cardiomyocyte hypertrophy (56). Metabolic signaling and mitochondrial dysfunction have been recognized as common pathogenic mechanisms in patients with HCM based on previous studies (57, 58). Specifically, the impact of CYBB on cardiac energetics and/or the progression of ventricular dysfunction has been reported (59). However, there is limited research on the role of CYBB in regulating altered cardiac energetics and mitochondrial dysfunction in HCM, as highlighted by the existing literature. Previous studies have shown that mice with a global deficiency in CYBB expression exhibit partial protection against the development of experimental autoimmune encephalomyelitis (EAE) following active immunization. However, there is limited research on the role of CYBB in modulating the immune microenvironment in HCM. Additional research is required to fully understand CYBB's potential mechanisms in HCM, including its impact on myocardial congenital development or its involvement in inflammatory processes triggered by environmental stimuli. In-depth research is needed to elucidate the underlying mechanisms implicated in the relationship between CYBB and HCM.

Due to our use of public datasets (GSE130036 and GSE141910) from the GEO database and an imbalance favoring a higher number of controls, limitations exist in interpreting our findings. Additionally, the two datasets are from heterogeneously composed populations with different composition such as gender, age and race which exhibit different distributions, potentially influencing the analysis conducted in this study. Secondly, although we performed a rigorous bioinformatics analysis of the transcriptome, validation at the protein level through independent experiments and clinical trials is necessary to confirm the results. It is noteworthy that the immune infiltration analysis, based on transcriptomic data using the CIBERSORT algorithm, does not establish causality between immune cells and necroptosis or their involvement in the process. Further investigations are required to elucidate the precise relationship between immune cells and necroptosis. Despite these limitations, our study contributes to the existing literature on necroptosis in HCM and provides valuable insights into potential diagnostic biomarkers and therapeutic targets for further exploration (33). However, there is limited research on the identification of gene biomarkers associated with immune infiltration and necroptosis in hypertrophic cardiomyopathy (HCM) compared to normal tissues. In this study, we identified 17 NRDEGs. Based on our analysis of the PPI network in GSE1360036, we identified nine hub genes: HSP90AA1, CYBB, STAT3, BIRC3, BCL2, PYCARD, STAT4, JAK2, and TLR4. GSEA analysis revealed several significant biological pathways that may contribute to HCM development. Our analysis of two GEO datasets revealed the potential diagnostic value of CYBB, BCL2, and JAK2 in HCM. Interestingly, there were significant differences in immune cell composition between HCM and normal samples. Specifically, CYBB showed a positive correlation with M2 macrophages, while BCL2 and JAK2 exhibited negative correlations. Despite these constraints, our study contributes towards the understanding of necroptosis in HCM and offers potential diagnostic biomarkers and therapeutic targets which worth further investigation.

## 5 Conclusions

Bioinformatics analyses discerned influential differences in necroptosis-associated gene expression amidst HCM and normal heart tissue samples. CYBB, BCL2, and JAK2 emerged as promising HCM diagnostic biomarkers. Further, our research discovered a relationship between M2 cell imbalance, expression of necroptosis-related genes, and infiltration by various immune cell types into HCM tissue. Collectively, these findings shed light on the underlying mechanisms of HCM pathophysiology, notably concerning necroptosis and immune factors, and pave the way for potential therapeutic HCM interventions.

## Data Availability

Publicly available datasets were analyzed in this study. This data can be found here: https://www.ncbi.nlm.nih.gov/geo/, GSE130036 and GSE141910.
